# Enhanced production of biomass and lipids by *Euglena gracilis* via co-culturing with a microalga growth-promoting bacterium, *Emticicia* sp. EG3

**DOI:** 10.1186/s13068-019-1544-2

**Published:** 2019-10-31

**Authors:** Tadashi Toyama, Tsubasa Hanaoka, Koji Yamada, Kengo Suzuki, Yasuhiro Tanaka, Masaaki Morikawa, Kazuhiro Mori

**Affiliations:** 1 Graduate Faculty of Interdisciplinary Research, University of Yamanashi, 4-3-11 Takeda, Kofu, Yamanashi 400-8511 Japan; 2 Integrated Graduate School of Medicine, Engineering, and Agricultural Sciences, University of Yamanashi, 4-3-11 Takeda, Kofu, Yamanashi 400-8511 Japan; 3 Euglena Co., Ltd., 5-29-11 Shiba Minato-ku, Tokyo, 108-0014 Japan; 4 Microalgae Production Control Technology Laboratory, RIKEN, 1-7-22, Suehiro, Tsurumi, Yookohama, Kanagawa 230-0045 Japan; 5 Division of Biosphere Science, Graduate School of Environmental Science, Hokkaido University, Kita-10 Nishi-5, Kita-ku, Sapporo, 060-0810 Japan

**Keywords:** *Euglena gracilis*, Growth-promoting bacteria, Biomass production, Lipid production, Wastewater effluent, Wastewater effluent

## Abstract

**Background:**

*Euglena gracilis*, a unicellular flagellated microalga, is regarded as one of the most promising species as microalgal feedstock for biofuels. Its lipids (mainly wax esters) are suitable for biodiesel and jet fuel. Culture of *E. gracilis* using wastewater effluent will improve the economics of *E. gracilis* biofuel production. Enhancement of the productivity of *E. gracilis* biomass is critical to creating a highly efficient biofuels production system. Certain bacteria have been found to promote microalgal growth by creating a favorable microenvironment. These bacteria have been characterized as microalgae growth-promoting bacteria (MGPB). Co-culture of microalgae with MGPB might offer an effective strategy to enhance microalgal biomass production in wastewater effluent culture systems. However, no MGPB has been identified to enhance the growth of *E. gracilis*. The objectives of this study were, therefore, to isolate and characterize the MGPB effective for *E. gracilis* and to demonstrate that the isolated MGPB indeed enhances the production of biomass and lipids by *E. gracilis* in wastewater effluent culture system.

**Results:**

A bacterium, *Emticicia* sp. EG3, which is capable of promoting the growth of microalga *E. gracilis*, was isolated from an *E. gracilis*-municipal wastewater effluent culture. Biomass production rate of *E. gracilis* was enhanced 3.5-fold and 3.1-fold by EG3 in the co-culture system using a medium of heat-sterilized and non-sterilized wastewater effluent, respectively, compared to growth in the same effluent culture but without EG3. Two-step culture system was examined as follows: *E. gracilis* was cultured with or without EG3 in wastewater effluent in the first step and was further grown in wastewater effluent in the second step. Production yields of biomass and lipids by *E. gracilis* were enhanced 3.2-fold and 2.9-fold, respectively, in the second step of the system in which *E. gracilis* was co-cultured with EG3 in the first step.

**Conclusion:**

*Emticicia* sp. EG3 is the first MGPB for *E. gracilis*. Growth-promoting bacteria such as EG3 will be promising agents for enhancing *E. gracilis* biomass/biofuel productivities.

## Background

Microalgae are widely recognized as alternative, renewable, and sustainable feedstocks for biofuels. The use of wastewater effluent as a medium for microalgal culture will no doubt improve the economics and sustainability of microalgal biomass production [1, 2]. Microalgal biomass production using wastewater effluent decreases the costs for fertilization and irrigation. Coupling microalgal production with wastewater treatment would transform a wastewater treatment plant into a biorefinery plant [3].

*Euglena gracilis*, a unicellular flagellated microalga, is regarded as one of the most promising species as microalgal feedstock for functional foods and biofuels [4, 5]. Depending on culture conditions, *E. gracilis* can synthesize and accumulate a variety of valuable products such as wax esters [6], paramylon (β-1,3-glucan) [7], β-carotene, and vitamins C and E [8]. Those products have numerous commercial applications. Wax esters—which consist mainly of C14:0 saturated fatty acid, myristic acid, C14:0 saturated fatty alcohols, and myristyl alcohols—are considered high-value biofuels [9, 10]. These wax esters of *E. gracilis* are suitable for biodiesel and jet fuel [5, 10, 11].

Enhancement of the productivity of *E. gracilis* biomass is critical to creating a highly efficient biofuels production system and to reducing the price of the biofuel. Genetic recombination [12], selective breeding [13], optimization of growth conditions [14, 15], and supplementation with additives, including nutrients [14] and plant hormones [16], have been investigated to enhance the production of biomass and lipids by *E. gracilis*. However, to realize a highly efficient *E. gracilis*’s biofuel producing system, development of synergistic technologies is needed to further enhance the growth of *E. gracilis*.

In both natural aquatic environments and microalgal cultures, certain bacteria have been found to promote microalgal growth by creating a favorable microenvironment [17–19], such as providing nutrients [20], vitamins [21], phytohormones [22], chelators [23], or volatile organic compounds [24]. These bacteria have been characterized as microalgae growth-promoting bacteria (MGPB). MGPB have been reported for various microalgae. For example, *Azospirillum brasilense* for *Chlorella vulgaris* [25]; *Rhizobium* sp. for *Botryococcus braunii* [26]; *Pelagibaca bacilliformis* and *Stappia* sp. for *Tetraselmis striata* [27]; and *Rhizobium* sp. for *Chlamydomonas reinhardtii*, *C. vulgaris*, *Scenedesmus* sp., and *B. braunii* [28]. The use of MGPB as inoculant in co-cultures with microalgae might offer an alternative and promising strategy to effectively enhance microalgal biomass production. To the best of our knowledge, however, no MGPB has been identified to enhance the growth of *E. gracilis*. In addition, no studies have shown that MGPB enhance microalgal biomass production in wastewater effluent under non-sterile culture conditions where exist indigenous bacteria. There is thus high demand for obtaining a novel MGPB that is capable of enhancing biomass/lipid production by *E. gracilis* in wastewater effluent.

The objectives of this study were, therefore, to (i) isolate and characterize the MGPB effective for *E. gracilis* and (ii) demonstrate that the isolated MGPB indeed enhances the production of biomass and lipids by *E. gracilis* in wastewater effluent culture system.

## Methods

### *E. gracilis* material and culturing of *E. gracilis*

Axenic *E. gracilis* (NIES-48) was obtained from the Microbial Culture Collection, National Institute for Environmental Studies, Tsukuba, Japan. *E. gracilis* was cultured in C medium supplemented with 400 mg/L yeast extract and 600 mg/L polypeptone, namely CYP medium. C medium contained per liter 150 mg Ca(NO_3_)_2_·4H_2_O, 100 mg KNO_3_, 50 mg β-Na_2_ glycerophosphate·5H_2_O, 40 mg MgSO_4_·7H_2_O, 500 mg tris(hydroxymethyl)aminomethane, 0.1 μg vitamin B_12_, 0.1 μg biotin, 10 μg thiamine HCl, and 3 mL PIV metals (1000 mg/L Na_2_EDTA·H_2_O, 196 mg/L FeCl_3_·6H_2_O, 36 mg/L MnCl_2_·4H_2_O, 10.4 mg/L ZnCl_2_, 4 mg/L CoCl_2_·6H_2_O, and 2.5 mg/L Na_2_MoO_4_·H_2_O). pH was adjusted to 7.5 by adding NaOH. The axenic *E. gracilis* culture was incubated in a growth chamber (28 ± 1 °C with fluorescent lamps at a photosynthetic photon flux density of 80 μmol/m^2^/s and 16-h photoperiod) for 1 week. Thereafter, subculture was started by routine transfer of *E. gracilis* cells into fresh CYP medium every week. *E. gracilis* was harvested by centrifugation (3000×*g*, room temperature, 5 min), washed with sterile C medium, and re-suspended in aliquots of sterile C medium. Such an *E. gracilis* cell suspension was used as the inoculum for each subculture experiment.

### Wastewater effluent samples

Municipal wastewater effluent was collected from the final sedimentation process of a conventional activated sludge municipal wastewater treatment plant in Kofu City, Yamanashi, Japan. The effluent sample was first passed through a glass microfiber filter (pore size, 1 μm; GF/B grade; GE Healthcare UK Ltd, Buckinghamshire, England) and then through a membrane filter (pore size, 0.8 μm; mixed cellulose esters membrane; Merck Millipore Ltd, Cork, Ireland) to remove suspended solids and organisms larger than bacteria—including microalgae—from the effluent sample. The effluent filtrate, therefore, included the indigenous bacterial community. For each of the following experiments, a fresh effluent sample was collected and used each day. Table 1 shows the water quality characteristics (total organic carbon [TOC], ammonium-nitrogen [NH_4_-N], nitrite-N [NO_2_-N], nitrate-N [NO_3_-N], phosphate [PO_4_-P]), and total bacteria concentrations). TOC was measured using TOC-LCSH (Shimadzu, Kyoto, Japan). For NH_4_-N, the indophenol method was used; for NO_2_-N, the *N*-(1-naphthyl) ethylenediamine method was used; for NO_3_-N, the reduction-*N*-(1-naphthyl) ethylenediamine method and UV adsorption (at 220 and 275 nm) method was used; and for PO_4_-P, the molybdenum blue method was used. Total culturable bacteria in effluent were quantified using R2A agar plates (0.5 g/L Peptone, 0.5 g/L Yeast extract, 0.5 g/L Casamino acid, 0.5 g/L Glucose, 0.5 g/L Soluble starch, 0.3 g/L K_2_HPO_4_, 0.05 g/L MgSO_4_·7H_2_O, 0.3 g/L Sodium pyruvate; pH 7.0; Agar 15 g/L). In some experiments, sterile effluent samples were prepared by autoclaving (121 °C, 20 min) for heat sterilization.

**Table 1:** Initial concentrations of total organic carbon (TOC), inorganic nitrogen (NH_4_-N, NO_2_-N, and NO_3_-N), phosphate (PO_4_-P), and total bacteria in effluent samples for each experiment

	TOC (mg/L)	NH_4_-N (mg/L)	NO_2_-N (mg/L)	NO_3_-N (mg/L)	PO_4_-P (mg/L)	Total bacteria (CFU/mL)
Experiment of section 2.3	15.4	7.2	0.0	6.1	3.3	4.5 ± 0.2 × 10^5^
Experiment of section 2.4	13.9	6.8	0.0	6.9	3.1	1.9 ± 0.1 × 10^5^
Experiment of section 2.7	14.2	6.1	0.0	5.8	3.4	3.6 ± 0.2 × 10^5^
Experiment of section 2.8	16.5	6.6	0.0	6.6	3.3	6.4 ± 0.5 × 10^5^

### Culturing of *E. gracilis* in wastewater effluent and isolation of MGPB candidates for *E. gracilis*

A cell suspension (10 mL) of *E. gracilis* prepared by the above method was added to 100 mL of (non-sterilized) effluent filtrate in a 200-mL glass flask. The flask was incubated in the growth chamber (28 ± 1 °C with fluorescent lamps at 80 μmol photons m^−2^ s^−1^ and 16-h photoperiod) for 10 days, and then, about 10 mL of this culture was transferred to 100 mL of fresh effluent and incubated for another 10 days. This transfer and 10-day batch culture growth was repeated once more. During the three repeated growth periods, the flasks were shaken for 1 min three times a day to disperse and aerate the *E. gracilis*. The chlorophyll concentration in each flask was measured daily. *E. gracilis* was also cultured in 100 mL of autoclaved effluent without living indigenous bacteria. The culture was used as a control treatment to determine the effect of the indigenous bacteria in the effluent on the growth of *E. gracilis*. The initial chlorophyll *a* + *b* concentration of each culture was adjusted to about 0.5 μg/mL.

After the third 10-day batch culture growth period, 20 mL of the non-sterile *E. gracilis*-effluent culture was transferred into a 50-mL tube, vortexed at maximum speed for 3 min, ultrasonicated (40 kHz) for 1 min, and vortexed again to disperse the *E. gracilis* and bacterial cells. The sample was then filtered through a GF/B glass microfiber filter to remove *E. gracilis* cells. The filtrate-containing bacteria was serially diluted and spread on R2A agar plates. The plates were then incubated at 28 °C. A total of ten bacterial strains were obtained from the isolation culture. A pure culture of each strain was maintained on an R2A agar plate.

### Screening of MGPB

Six isolated strains grew well in R2A liquid medium. Each strain was cultured in R2A liquid medium for 24 h at 28 °C. Cells were harvested by centrifugation (10,000×*g*, room temperature, 5 min) and washed twice with 50 mM potassium phosphate buffer (pH 7.5). *E. gracilis* was inoculated into 100 mL of autoclaved wastewater effluent in 200-mL glass flasks. Optical densities of cultures of isolated bacterial cells were measured at 600 nm (OD_600_), and each bacterial cell suspension was then added into the *E. gracilis* culture at bacterial cell density at OD_600_ of 0.05. The co-cultures of *E. gracilis* and each isolated strain were incubated in a growth chamber (28 ± 1 °C with fluorescent lamps at 80 μmol photons/m^2^/s and 16-h photoperiod) for 7 days. At 7th day, concentrations of chlorophyll *a* + *b* were measured. *E. gracilis* was also cultured in autoclaved wastewater effluent without a bacterial inoculation. The growth-promoting ability of MGPB was assessed by comparing the chlorophyll *a* *+ b* concentration at the end of 7-day cultures with and without bacterial inoculation.

### Identification and characterization of strain EG3

Among the isolated bacterial strains, strain EG3 showed the highest growth-promoting ability in the screening test. Strain EG3 was thus characterized and identified by physiological and phylogenetic analyses. The physiological characterization was performed with an API 20NE kit according to the manufacturer’s instructions (BioMérieux Japan, Tokyo, Japan). A comparative 16S rRNA gene sequence analysis was performed as follows. Partial 16S rRNA genes were amplified by PCR using primers 8F (5′-AGAGTTTGATCCTGGCTCAG-3′) [29] and 1510R (5′-GGTTACCTTGTTACGACTT-3′) [30]. A genus-level identification was carried out based on 16S rRNA gene sequence similarity with that of a type strain sequence in NCBI-GenBank using BLAST. The 16S rRNA sequence data (1431 bp) of isolated strain EG3 have been submitted to the DDBJ/EMBL/GenBank databases under accession number LC441033.

### Culture conditions of EG3 and preparation of cell suspension

Strain EG3 was grown in R2A liquid medium for 24 h at 28 °C with shaking at 150 rpm. Cells were harvested by centrifugation (10,000×*g*, room temperature, 5 min), washed twice with phosphate buffer, and then suspended in the phosphate buffer. Highly concentrated EG3 cell suspension in phosphate buffers (DO_600_ = 5, 10, or 20) were prepared for the following experiments. For each experiment, 1% (v/v) of the EG3 cell suspension was inoculated into the experimental culture (sterilized or non-sterilized effluent). Thus, the initial EG3 cell density of each experiment was adjusted as OD_600_ = 0.05, 0.1, or 0.2. To convert OD_600_ value into EG3 cell dry weight concentration (mg dry weight/mL) and cell count (CFU/mL), dry weight of cells was measured after drying at 90 °C for 3 h, and CFU of EG3 suspensions (OD_600_ = 0.05, 0.1, and 0.2) was measured by R2A agar plate method in triplicate. An OD_600_ = 1.0 of EG3 cells was equivalent to about 0.45 ± 0.03 mg dry weight/mL. Also, OD_600_ = 0.05, 0.1, and 0.2 of EG3 cells were equivalent to about 2 × 10^6^, 8 × 10^6^, and 4 × 10^7^ CFU/mL on R2A agar plate. In this manuscript, cell density of EG3 was described using CFU/mL.

### Co-culturing of *E. gracilis* with EG3 in effluent under several conditions

*Euglena gracilis* was inoculated into 1 L of autoclave sterilized wastewater effluent in a 2-L glass bottle. To determine the effect of the EG3 cell density on the growth of *E. gracilis*, EG3 was inoculated into 1 L of an *E. gracilis* culture in sterile wastewater effluent at an initial cell density of 2 × 10^6^, 8 × 10^6^, and 4 × 10^7^ CFU/mL. The co-cultures of *E. gracilis* with EG3 were incubated in a growth chamber (28 ± 1 °C with fluorescent lamps at 80 μmol photons/m^2^/s and 16-h photoperiod) for 7 days. *E. gracilis* was also cultured without EG3 in 1 L of autoclave sterilized wastewater effluent under the above conditions as the control experiment.

Both sterilized and non-sterilized effluent samples were used to examine the effects of co-existing indigenous bacteria in wastewater effluent on the ability of EG3 to promote *E. gracilis* growth. *E. gracilis* was inoculated into 1 L of wastewater effluent in 2-L glass bottles. EG3 was then inoculated into the *E. gracilis* culture at 8 × 10^6^ CFU/mL, because the growth-promoting ability of EG3 was highest at this inoculation cell density. The co-cultures of *E. gracilis* with EG3 were incubated in the growth chamber (28 ± 1 °C with fluorescent lamps at 80-μmol photons/m^2^/s and 16-h photoperiod) for 7 days.

During the experiments, chlorophyll *a* + *b* concentration was monitored daily. After 7 days, the dry weight of *E. gracilis* was measured. The experiments were conducted in triplicate.

### Experiment to demonstrate EG3-enhanced production of biomass and lipids by *E. gracilis* in wastewater effluent: two-step enhanced *E. gracilis* biomass/lipids production system

**Fig. 1:**
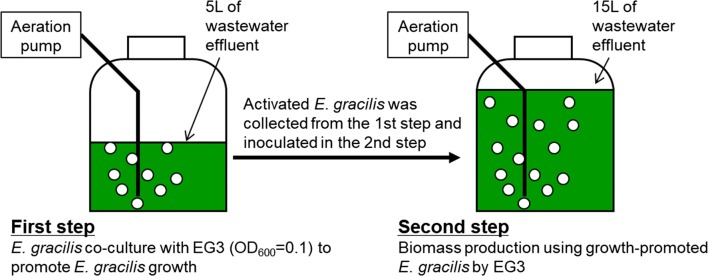
Schematic design of two-step enhanced *E. gracilis* biomass/lipid production system

In this part of our study, possibility of a two-step enhanced biomass production system was evaluated. The first step was co-culture of *E. gracilis* with strain EG3 to promote the growth of *E. gracilis*. The *E. gracilis* cells produced in this first step were used as the *E. gracilis* inoculum for the second step. In the second step, the productions of biomass and lipids by the *E. gracilis* inoculum from the first step were measured (Fig. 1).

The experimental design for the co-culture of *E. gracilis* and EG3 in the first step was as follows. *E. gracilis* was inoculated into 5 L of non-sterilized wastewater effluent in a 15-L polycarbonate culture vessel with an inner diameter of about 290 mm and a height of about 430 mm. EG3 was inoculated into the *E. gracilis* culture at 8 × 10^6^ CFU/mL. The co-culture of *E. gracilis* and EG3 in the first step was bubbled with atmospheric air by an air pump at a flow rate 0.5 L/min for 7 days in a growth chamber (28 ± 1 °C with fluorescent lamps at 80-μmol photons/m^2^/s and 16-h photoperiod). After 7 days, 2–4 L of culture was collected, and *E. gracilis* was harvested by low gravity centrifugation (3000×*g*, 15 min). The *E. gracilis* cells were washed twice with distilled water to remove EG3 cells and harvested by centrifugation. We confirmed that the collected *E. gracilis* pellets contained only little EG3 cells (Additional file 1: Fig. S1). The collected *E. gracilis* pellet was used for the second step culture. *E. gracilis* was also cultured without EG3 in 5 L of wastewater effluent under the above conditions, and the control *E. gracilis* pellet from this experiment was also used for the second step.

The production of biomass and lipids in the second step culture was performed in 15-L polycarbonate culture vessels. Fifteen liters of unautoclaved wastewater effluent was added to the culture vessel. The *E. gracilis* co-cultured with EG3 or the control *E. gracilis* only culture was inoculated into the unautoclaved wastewater effluent. The initial cell density of *E. gracilis* was about 80 mg dry weight/L. The biomass/lipid production step was conducted in a growth chamber (28 ± 1 °C with fluorescent lamps at 80 μmol photons/m^2^/s and 16-h photoperiod) for 7 days. Aeration and mixing were achieved by bubbling with atmospheric air at a flow rate 1 L/min. Chlorophyll *a* + *b* and biomass were measured every day. The lipid content of *E. gracilis* was measured at the beginning and end of the second step.

### *E. gracilis* growth measurement and *E. gracilis* lipid measurement

In this study, chlorophyll concentration was monitored as an indicator of *E. gracilis* growth. Chlorophyll concentration was measured spectrophotometrically after extraction in 100% methanol for 30 min [31]. Absorbance of the extract was measured at 665 nm (A_665_) and 650 nm (A_650_) with a spectrophotometer (UVmini-1240; Shimadzu Co. Ltd., Kyoto, Japan). The total chlorophyll (chlorophyll *a* + chlorophyll *b*: Chl *a* + *b*) concentration (μg/mL) was calculated as follows [31]:

1 $${\text{Chl }} a + b\;(\upmu{\text{g/mL}}) = 4\times {\text{A}}_{ 6 6 5} + 2 5. 5\times {\text{A}}_{ 6 50} .$$

The biomass (dry weight) of *E. gracilis* was measured as follows. Fifty milliliters of the culture was collected and vortexed for 30 s to uniformly suspend the bacterial and microalgal cells. The mixture was centrifuged (3000×*g*, 5 min); the pellet was washed with 20 mL of distilled water; and the pellet was then suspended in 20 mL of distilled water. The *E. gracilis* cells in the suspension were collected on a pre-weighed GF/B filter (pore size, 1 μm), dried (90 °C, 3 h), and then weighed. We confirmed that this method could collect *E. gracilis* cells with little interference from co-existing EG3 cells (Additional file 1: Fig. S1).

The lipid content of the *E. gracilis* cells was quantified in terms of the percent of dry biomass accounted for by lipids. *E. gracilis* was harvested by centrifugation (3000×*g*, 5 min), washed with distilled water, dried, and then powdered. The *E. gracilis* powder (20 mg) was crushed with 1 mL of *n*-hexane in a BioMasher (Takara Bio, Kusatsu, Japan). The *E. gracilis* sample was transferred into a 50-mL tube, and 9 mL of *n*-hexane and 6-mL isopropanol were added to the tube. The tube was shaken at 225 rpm for 24 h. Thirty milliliters of distilled water was then added to the tube. The tube was shaken for 1 min and then centrifuged (10,000×*g*, 5 min). The *n*-hexane layer containing the lipids was collected on a pre-weighed aluminum tray, dried at room temperature overnight, and then dried at 90 °C for 3 h. Finally, the collected lipids were weighed.

Biomass production rate (mg/L/day) and lipid production rate (mg/L/day) were calculated as follows:

2 $${\text{Biomass production rate (mg/L/day)}} = [ {\text{final biomass (mg/L)}}{-}{\text{initial biomass (mg/L)]/cultivation time (day)}}$$

3 $${\text{Lipid production rate (mg/L/day)}} = {\text{final biomass (mg/L)}} \times {\text{final lipid content (}}\% ){-}{\text{initial biomass (mg/L)}} \times {\text{initial lipid content}}(\%) / {\text{cultivation time (day)}}.$$

### Statistical analysis

Each value used in the statistical analysis represents the results from three samples (*n* = 3 replicates) per experiment. All results are expressed as mean ± SD. Significance (*p* < 0.05) was analyzed using the *t* test in SPSS Statistics v. 22.0 (IBM, Armonk, NY, USA).

## Results

### Isolation and identification of the MGPB *Emticicia* sp. EG3 for *E. gracilis*

**Fig. 2:**
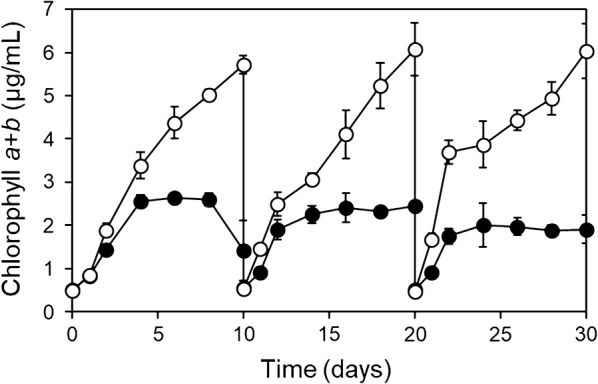
Changes in chlorophyll *a* + *b* content in *E. gracilis*-municipal wastewater effluent culture with indigenous living bacteria (non-sterilized effluent, white circle) and without living indigenous bacteria (autoclave heat-sterilized effluent, black circle). Values are means ± SDs (*n* = 3)

To test the hypothesis that indigenous bacteria in wastewater effluent could enhance the growth of *E. gracilis*, *E. gracilis* was grew in wastewater effluent with (non-sterilized effluent) or without living bacteria (autoclave heat-sterilized effluent) in three sequencing batch cultures. After 2 days of each batch culture, the chlorophyll *a* + *b* concentration was higher (*p* < 0.05) in the *E. gracilis*-effluent culture with living indigenous bacteria than in the culture without living bacteria (Fig. 2). The results suggested that MGPB were present in the *E. gracilis*-effluent culture with living indigenous bacteria. Ten bacterial strains were isolated from the *E. gracilis* culture in non-sterilized effluent. Six of the ten strains grew well in R2A medium and were examined for their ability to enhance *E. gracilis* growth. Four strains were found to enhance the growth of *E. gracilis*. Among them, strain EG3 showed the highest growth-promoting ability (Additional file 2: Fig. S2). In addition, strain EG3 showed high biomass yield in R2A liquid medium culture.

Colonies of strain EG3 on R2A agar were circular, smooth, convex, and orange color. EG3 showed positive for oxidase, glucose fermentation, and aesculin hydrolysis, but negative for nitrate reduction, indole production, arginine dihydrolase, urease, gelatinase, and β-galactosidase activities. EG3 utilized glucose, d-mannose, or maltose as sole carbon sources but did not utilize l-arabinose, d-mannitol, *N*-acetyl-d-glucosamine, gluconate, *n*-caprate, adipate, d,l-malate, citrate, or phenylacetate. Almost the entire sequence of the 16S rRNA gene (1431 bp) of EG3 was very similar to the analogous sequences of *Emticicia fontis* strain IMCC1731^T^ (97.9% sequence similarity), *Emticicia ginsengisoli* strain Gsoil 085^T^ (97.6%), *Emticicia soli* strain ZZ-4^T^ (97.5%), *Emticicia oligotrophica* strain GPTSA100-15^T^ (94.4%), *Emticicia aquatica* strain HMF2925^T^ (93.9%), *Emticicia sediminis* strain JBR12^T^ (93.7%), and *Emticicia aquatilis* strain THG-DN6.14^T^ (93.7%). We, therefore, identified strain EG3 as *Emticicia* sp.

### Growth promotion of *E. gracilis* by strain EG3 at different cell densities

**Fig. 3:**
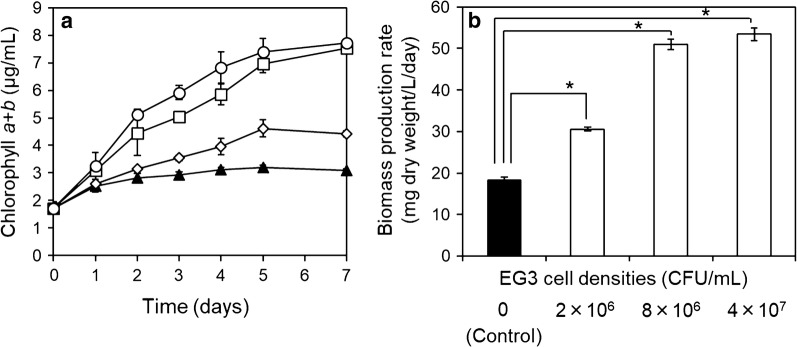
Effects of EG3 on the growth of *E. gracilis* co-cultured with various initial cell densities of EG3—0 (control), 2 × 10^6^, 8 × 10^6^, and 4 × 10^7^ CFU/mL—in heat-sterilized wastewater effluent for 7 days. **a** Changes in chlorophyll *a* + *b* content in *E. gracilis* co-cultured with EG3 at initial cell density of 0 (control, black triangle), 2 × 10^6^ CFU/mL (white diamond), 8 × 10^6^ CFU/mL (white square), or 4 × 10^7^ CFU/mL (white circle). **b** Biomass production rate of *E. gracilis* in co-culture with EG3 at initial cell density of 0 (control), 2 × 10^6^, 8 × 10^6^, and 4 × 10^7^ CFU/mL during 7 days. Values are means ± SDs (*n* = 3). *Significant difference (*p* < 0.05) from control

To determine the cell density of EG3 needed to promote the growth of *E. gracilis* in wastewater effluent, *E. gracilis* was grew in heat-sterilized wastewater effluent with various EG3 cell densities, 0 (control), 2 × 10^6^, 8 × 10^6^, and 4 × 10^7^ CFU/mL. The chlorophyll *a* + *b* concentrations and biomass production rate (mg/L/day) of *E. gracilis* were significantly higher (*p* < 0.05) in *E. gracilis* cultures with EG3 at 2 × 10^6^, 8 × 10^6^, and 4 × 10^7^ CFU/mL than in the *E. gracilis* control culture (Fig. 3). The growth-promoting effect of EG3 was higher at cell density of 8 × 10^6^ and 4 × 10^7^ CFU/mL than at 2 × 10^6^ CFU/mL. The EG3 initial inoculation cell density was an important determinant of whether EG3 greatly enhanced *E. gracilis* biomass production rate. We decided to use 8 × 10^6^ CFU/mL for follow-up experiments, because the difference in the ability of EG3 to promote the growth of *E. gracilis* between initial inocula 8 × 10^6^ and 4 × 10^7^ CFU/mL was not significant.

### Effect of indigenous bacteria in wastewater effluent on the ability of EG3 to promote the growth of *E. gracilis*

**Fig. 4:**
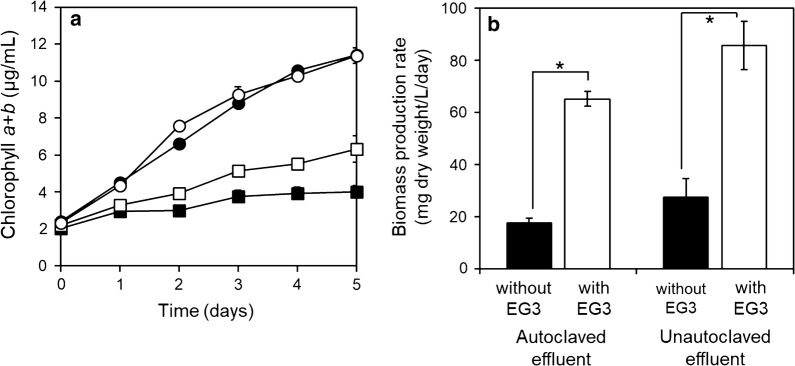
Effects of EG3 on the growth of *E. gracilis* co-cultured with initial EG3 cell density of 8 × 10^6^ CFU/mL in heat-sterilized and non-sterilized wastewater effluents for 7 days. **a** Changes in chlorophyll *a* + *b* content in *E. gracilis* cultured without (black square) and with EG3 (white square) in heat-sterilized effluent; changes in chlorophyll *a* + *b* content in *E. gracilis* cultured without (black circle) and with EG3 (white circle) in non-sterilized effluent. **b** Biomass production rate of *E. gracilis* cultured without or with EG3 in sterilized or non-sterilized effluent during 7 days. Values are means ± SDs (*n* = 3). *Significant difference (*p* < 0.05) from control

To examine the effect of bacteria that co-existed with strain EG3, *E. gracilis* and EG3 were co-cultured in heat-sterilized or non-sterilized wastewater effluent. The chlorophyll *a* + *b* concentrations of *E. gracilis*-EG3 co-cultures in both sterilized and non-sterilized wastewater effluent were significantly higher (*p* < 0.05) than those of *E. gracilis* cultures without EG3 (Fig. 4). During the 7-day culture experiment, the biomass production rate (mg/L/day) of *E. gracilis* co-cultured with EG3 were 3.7-fold and 3.1-fold in sterilized and non-sterilized wastewater effluent, respectively, those of *E. gracilis* cultured without EG3 (Fig. 4). These results clearly indicated that EG3 promoted the growth of *E. gracilis* in real wastewater effluent, which harbors a 3.6 ± 0.2 × 10^5^ CFU/mL complex indigenous bacterial community.

### Enhanced production of biomass and lipids by *E. gracilis* in effluent culture using strain EG3 in a two-step culture system

**Fig. 5:**
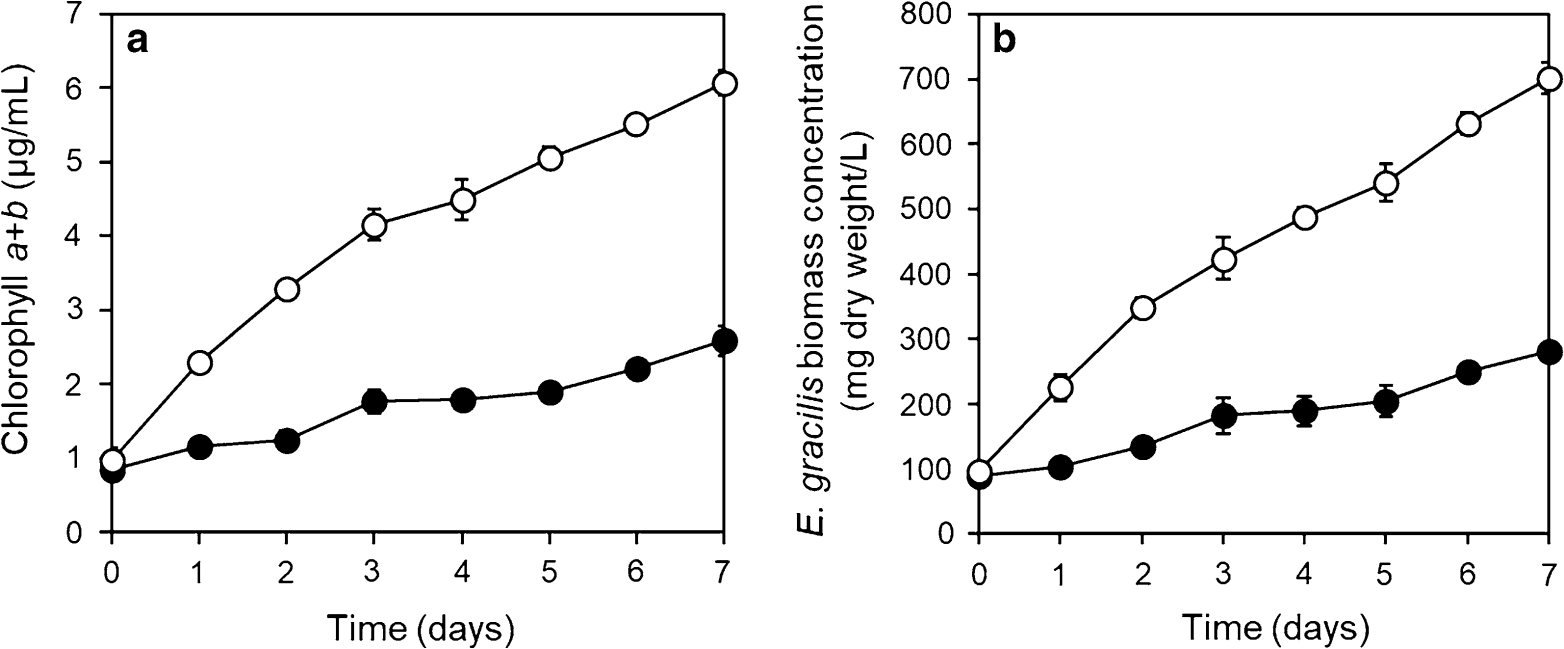
Time-course of chlorophyll *a* + *b* content and biomass (dry weight) concentration of *E. gracilis* in second culture of two-step *E. gracilis* culture systems. **a** Changes in chlorophyll *a* + *b* content in control culture of *E. gracilis* without co-culture with EG3 (black circle) and with co-culture with EG3 (white circle) in non-sterilized effluent during period of 7 days. **b** Changes in biomass concentration of *E. gracilis* in control culture without co-culture with EG3 (black circle) and with co-culture with EG3 (white circle) in non-sterilized effluent during period of 7 days. Values are means ± SDs (*n* = 3)

**Fig. 6:**
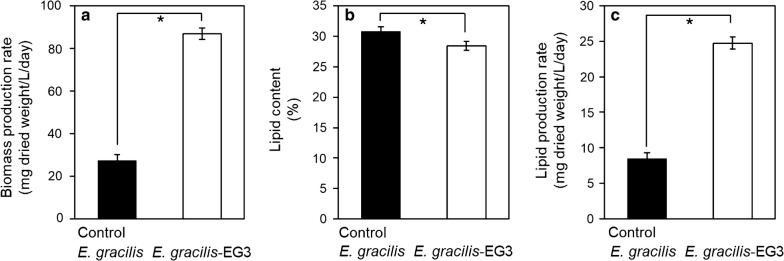
Effects of co-culturing *E. gracilis* with EG3 in first culture of a two-step *E. gracilis* biomass/lipid production system. **a** Biomass production rate of *E. gracilis* in second culture of two-step *E. gracilis* culture systems after co-culturing with EG3 and without (control) in the first step. **b** Lipid content of *E. gracilis* in second culture of two-step *E. gracilis* culture systems after co-culturing with EG3 and control. **c** Lipid production rate of *E. gracilis* in second culture of two-step *E. gracilis* culture systems after co-culturing with EG3 and control. Values are means ± SDs (*n* = 3). *Significant difference (*p* < 0.05) from control

We operated a two-step *E. gracilis* culture system to enhance production of biomass and lipids by *E. gracilis* (Fig. 1). First, *E. gracilis* was co-cultured with EG3 to promote *E. gracilis* growth. The *E. gracilis* produced in this first step was used as the inoculum for the second culture system, in which production of biomass and lipids by *E. gracilis* was monitored in 15 L of wastewater effluent for 7 days. In the control culture, *E. gracilis* was grown without EG3 in the first step, and then, the *E. gracilis* produced in the first step was used to inoculate a second culture system containing 15 L of wastewater effluent. The chlorophyll *a* + *b* concentration and biomass (dry weight) concentration of *E. gracilis* in the second culture system were significantly higher (*p* < 0.05) in the system inoculated with *E. gracilis* that had been co-cultured with EG3 in the first step than in the system inoculated with the control *E. gracilis* culture (Fig. 5). During the 7 days of second culture inoculated with the *E. gracilis*-EG3, *E. gracilis* biomass concentration reached the highest level of 702 ± 23 mg/L at the end of 7-day culture (Fig. 5). Figure 6 compares the rates of biomass and lipid production and the lipid content of *E. gracilis* during the second step in the systems inoculated with the *E. gracilis*-EG3 and control *E. gracilis* cultures. The rate of biomass production by *E. gracilis*-EG3 (87 ± 2.8 mg/L/day) was 3.2 times of that of the control *E. gracilis* (27 ± 2.7 mg/L/day). The lipid content of *E. gracilis*-EG3 (28.4% ± 0.7%) was slightly but significantly lower than that of the control *E. gracilis* (30.9% ± 0.8%). The lipid production rate of *E. gracilis*-EG3 (25 ± 0.8 mg/L/day) was 2.9 times that of the control *E. gracilis* (8.5 ± 0.8 mg/L/day).

## Discussion

Co-culture with MGPB is useful for enhancing microalgal growth in mass culture systems. For example, “*Candidatus* Phycosocius bacilliformis” BOTRYCO-2 and *P. bermudensis* KCTC 13073BP increase the rate of biomass production by *B. braunii* and *T. striata* 1.8-fold [32] and twofold [27], respectively. To make this strategy most successful, it is necessary to use the MGPB suitable for the host microalgal species. Use of the MGPB isolated from an *E. gracilis*-wastewater effluent culture was important for enhancing the biomass/lipid productivity of *E. gracilis* in a wastewater effluent culture system. The MGPB for three microalgae—*C. reinhardtii*, *C. vulgaris*, and *E. gracilis*—have recently been found to be present in common wastewater effluent [33]. In this study, an MGPB for *E. gracilis*, *Emticicia* sp. EG3, was isolated from an *E. gracilis*-municipal wastewater effluent culture and was found to be capable of promoting the growth of *E. gracilis*. Strain EG3 is the first MGPB for *E. gracilis*. Members of the genus *Emticicia* have been isolated from various environments, including freshwater [34–37], wetlands [38], seawater [39], the sediments of a shallow stream [40], and soil [41, 42]. Although the ecological role of *Emticicia* and its potential for industrial use are still unclear, our results reveal that one of its ecosystem services may be to function in a symbiotic relationship that enhances the growth of microalgae such as *E. gracilis*.

In co-cultures of *E. gracilis* with strain EG3 at 8 × 10^6^ CFU/mL, EG3 enhanced the production rate of *E. gracilis* biomass 3.7-fold and 3.1-fold in heat-sterilized and non-sterilized wastewater effluent, respectively (Fig. 4). The growth-promoting effect of EG3 on *E. gracilis* was similar to the effect of other MGPB on their host microalgae. These effects have previously been studied under single-MGPB and single-microalga axenic culture conditions without other bacterial communities. In natural field conditions, the introduction of plant growth-promoting bacteria often fails to show beneficial effects on the crops because of competition with other indigenous microorganisms [43, 44]. It is, therefore, noteworthy that strain EG3 significantly increased biomass productivity of *E. gracilis* in non-sterilized wastewater effluent, which harbors a complex community of indigenous bacteria. This ability of EG3 may, therefore, be of great practical value.

Some MGPB can increase the content of lipids [24, 45–47], fatty acids [47], starch [48], or hydrocarbons [32] in microalgal cells. *A. brasilense* Cd can significantly increase the accumulation of fatty acids and lipids in *C. vulgaris* [45, 47]. In this study, however, strain EG3 did not have any positive effect on the accumulation of lipids in *E. gracilis* (Fig. 6). The effects of EG3 on the production of other cellular components such as paramylon, β-carotene, starch, and proteins, or on lipid composition were not considered. Elucidation of those effects and that of the growth-promoting mechanisms of strain EG3 remain to be investigated.

Co-immobilization of a microalga-MGPB symbiosis has been proposed as a promising and effective strategy for enhancing microalgal abilities and for use in wastewater treatment and biomass production [49]. *C. vulgaris* has been co-immobilized with *A. brasilense* Cd in alginate beads, and the symbiosis has resulted in (1) enhanced nutrient removal in synthetic wastewater compared to immobilized *C. vulgaris* alone, and (2) enhanced biomass, lipid, and starch productivities in synthetic media [45, 48, 50]. In this study, we proposed a two-step culture system as another application of MGPB to enhance biomass production of *E. gracilis*. *E. gracilis* was first co-cultured with strain EG3 at 8 × 10^6^ CFU/mL to promote the growth of *E. gracilis*, and the production of biomass and lipids was then enhanced by growing the stimulated *E. gracilis* in wastewater effluent culture. In the second *E. gracilis* culture step, *E. gracilis* rapidly grew over a period of 7 days (Fig. 5). Biomass and lipid production rates during the 7-day culture period enhanced 3.2-fold and 2.9-fold, respectively, in real wastewater effluent compared to control *E. gracilis* cultures without EG3 inoculation (Fig. 6).

**Fig. 7:**
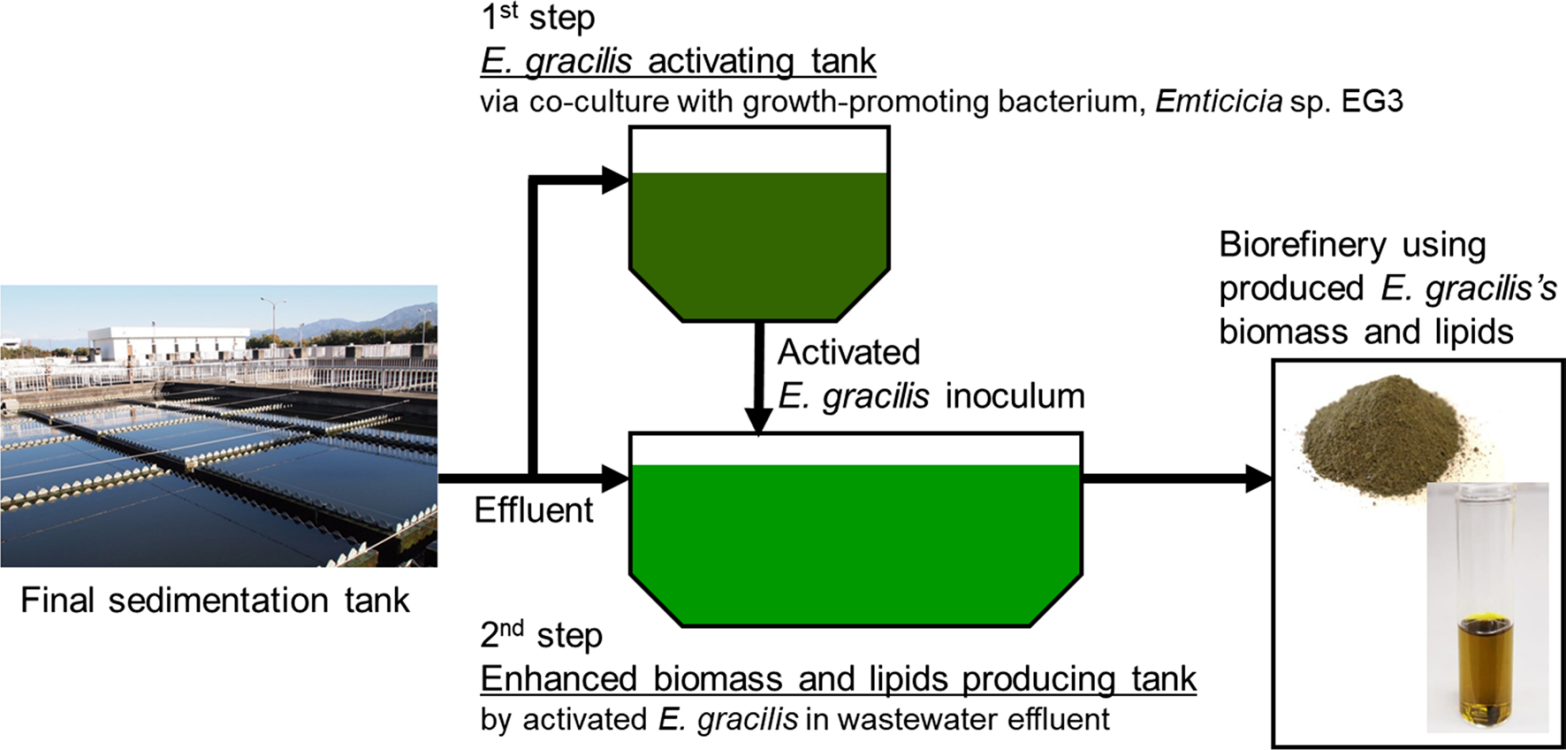
Coupling of *E. gracilis* culture with wastewater treatment. Enhanced biomass and lipid production by a two-step *E. gracilis* culture system using growth-promoting bacterium EG3

Our study is the first to demonstrate the potential of the MGPB *Emticicia* sp. EG3 to increase biomass/lipid productivities in wastewater-*E. gracilis* cultures. Promotion of *E. gracilis* growth has been achieved by genetic engineering or by supplements of exogenous nutrients, plant hormones, and ferulic acid. Ogawa et al. [12] were able to enhance *E. gracilis* biomass (dry weight) production rate twofold in CM medium under high-light and high CO_2_ conditions by overexpressing the cyanobacterial fructose-1,6-/sedoheptulose-1,7-bisphosphatase gene, which is involved in the Calvin cycle. The highest biomass concentration of the transgenic *E. gracilis* was 631.1 ± 89.9 mg/L. Zhu and Wakisaka [51] have reported that the addition of ferulic acid made from rice bran into an *E. gracilis* culture at 500 mg/L can increase *E. gracilis* cell density 2.5-fold and *E. gracilis* biomass (dry weight) production rate 2.2-fold. The maximum *E. gracilis* biomass concentration in CM medium with 500 mg/L ferulic acid was 670 ± 40 mg/L. Noble et al. [16] have reported that exogenous phytohormones stimulate the growth of *E. gracilis*; a combination of trans-zeatin (10^−7^ M) and abscisic acid (10^−9^ M) produced optimal conditions for growth of *E. gracilis* and increased growth rate about threefold compared to an *E. gracilis* culture without any phytohormones. Enhancement of biomass production rate (3.2-fold) and highest biomass concentration (702 ± 23 mg/L) of *E. gracilis* by EG3 co-culture at 8 × 10^6^ CFU/mL in real wastewater effluent were comparable to or higher than the results of these previous enhancement methods. Our results indicate the potential utility of a two-step *E. gracilis* culture system with EG3—the first step being co-culture of *E. gracilis* with EG3, and the second step being a large biomass production culture system for enhanced productivities of biomass and lipids by *E. gracilis* in wastewater effluent. A schematic design of the two-step *E. gracilis* culture system coupling with wastewater treatment plant is shown in Fig. 7. In combination with the other methods noted above, our strategy would greatly enhance the biomass/lipid productivities of *E. gracilis*. Optimization of the two-step culture system and better understanding of the growth-promoting effect of EG3 are the next challenges for commercial application of this strategy.

## Conclusions

*Emticicia* sp. EG3, which is capable of promoting the growth of *E. gracilis*, was isolated from an *E. gracilis*-municipal wastewater effluent culture. In a co-culture of EG3 and *E. gracilis*, EG3 significantly increased the biomass productivity of *E. gracilis*, not only in heat-sterilized wastewater effluent, but also in non-sterilized effluent that contained an indigenous wastewater effluent bacterial community. A two-step *E. gracilis* culture system was proposed to enhance *E. gracilis* biomass/lipid productivities by exploiting the stimulatory effect of EG3 on *E. gracilis*. First, *E. gracilis* was co-cultured with EG3 to promote *E. gracilis* growth in 5 L of effluent. Second, biomass and lipids were produced using the promoted *E. gracilis* in a 15-L wastewater effluent culture. Biomass and lipid productivities of *E. gracilis* were increased 3.2-fold and 2.9-fold, respectively, compared to a culture without EG3. Growth-promoting bacteria such as EG3 are promising agents for enhancing microalgal biomass/biofuels productivities.

## Supplementary information

**Additional file 1: Figure S1**. Summary of *E. gracilis* collection method and fate of EG3 cells during this method.

**Additional file 2: Figure S2**. Effects of isolated strains EG3, EG5, EG6, EG8, EG9, and EG10 on *E. gracilis* growth in autoclave heat-sterilized wastewater effluent.

## Data Availability

The data sets used and/or analyzed in this study are available from the corresponding author upon reasonable request.

## Abbreviations

MGPB: microalgae growth-promoting bacteria
TOC: total organic carbon
NH_4_-N: ammonium-nitrogen
NO_2_-N: nitrite-nitrogen
NO_3_-N: nitrate-nitrogen
PO_4_-P: phosphate
CFU: colony-forming units
OD: optical density
rRNA: ribosomal ribonucleic acid
PCR: polymerase chain reaction
NCBI: National Center for Biotechnology Information
BLAST: Basic Local Alignment Search Tool
DDBJ: DNA Data Bank of Japan
EMBL: European Molecular Biology Laboratory
SD: standard deviation
