# Supplementary material for: Enhanced production of biomass and lipids by Euglena gracilis via co-culturing with a microalga growth-promoting bacterium, Emticicia sp. EG3
Source: Biotechnol Biofuels. 2019 Oct 31;12:205. doi: 10.1186/s13068-019-1544-2 (PMC6822413; doi:10.1186/s13068-019-1544-2)
Supplement: Supplementary file 1 — Additional file 1: Figure S1. Summary of E. gracilis collection method and fate of EG3 cells during this method. [file 13068_2019_1544_MOESM1_ESM.docx]

Additional file 1

In this study, we examined the *E. gracilis* biomass collection method as follows. *E. gracilis* was pre-cultured in CYP medium in a growth chamber (28 ± 1 °C with fluorescent lamps at a photosynthetic photon flux density of 80 μmol m^−2^ s^−1^ and 16-h photoperiod) with shaking (120 rpm) for one week. *E. gracilis* cells were harvested by centrifugation (3000 × *g*, room temperature, 5 min) and washed with sterile C medium. And then, *E. gracilis* was inoculated into 1 L of autoclave sterilized wastewater effluent in a 2-L glass bottle.

Strain EG3 was pre-cultured in R2A liquid medium at 28 °C with shaking (150 rpm) for 24h. EG3 cells were harvested by centrifugation (10,000 × *g*, room temperature, 5 min), washed with sterile phosphate buffer, and then suspended in phosphate buffer. EG3 was inoculated into 1 L of the *E. gracilis* culture in sterile wastewater effluent at cell density of 1.11 ± 0.19× 10^7^ (mean ± SD) CFU/mL. The *E. gracilis*-EG3 culture was shaken at 120 rpm for 30 min.

Fifty milliliter of the culture was collected from the *E. gracilis*-EG3 culture and vortexed for 30 s to uniformly suspend the *E. gracilis* and EG3 cells. The mixture was centrifuged (3000 × *g*, 5 min; first centrifugation). The *E. gracilis* pellet was washed with 20 mL of sterile distileled water and 30 s vortex, and subjected to centrifugation (3000 × g, 5 min; second centrifugation) to remove EG3 cells. The *E. gracilis* pellet was suspended in 70 mL of sterile distilled water. The *E. gracilis* suspension (70 mL) was vortexed at maximum speed for 3 min, ultrasonicated (40 kHz) for 1 min, and vortexed again. The sample was then filtered through a GF/B glass microfiber filter to remove *E. gracilis* cells. The filtrate (70 mL) was serially diluted and spread on R2A agar plates. On the other hand, supernatant samples (70 mL) from the above first (50 mL) and second (20 mL) centrifugations were collected and mixed well. The supernatant sample (70 mL) was serially diluted and spread on R2A agar plates. The plates were then incubated at 28 °C, and EG3 colonies were counted. Finally, EG3 CFU/mL values obtained from *E. gracilis* pellet suspension and supernatant sample were multiplied by the dilution rate (70 mL/50 mL). The experiments were conducted in triplicate.

Summary of *E. gracilis* collection method and influence of EG3 on *E. gracilis* collection are shown in Fig. S1. Cell density of *E. gracilis* pellet suspension was 1.60 ± 0.28 × 10^5^ CFU/mL. The collected EG3 was 1.4% of the initial EG3 (1.11 ± 0.19 × 10^7^ CFU/mL) in *E. gracilis*-EG3 culture. On the other hand, EG3 cell density of supernatant sample was 1.05 ± 0.10 × 10^7^ CFU/mL. The collected EG3 was 94.6% of the initial EG3 in *E. gracilis*-EG3 culture. The results suggested that the collected *E. gracilis* pellet contained only little EG3 cells.


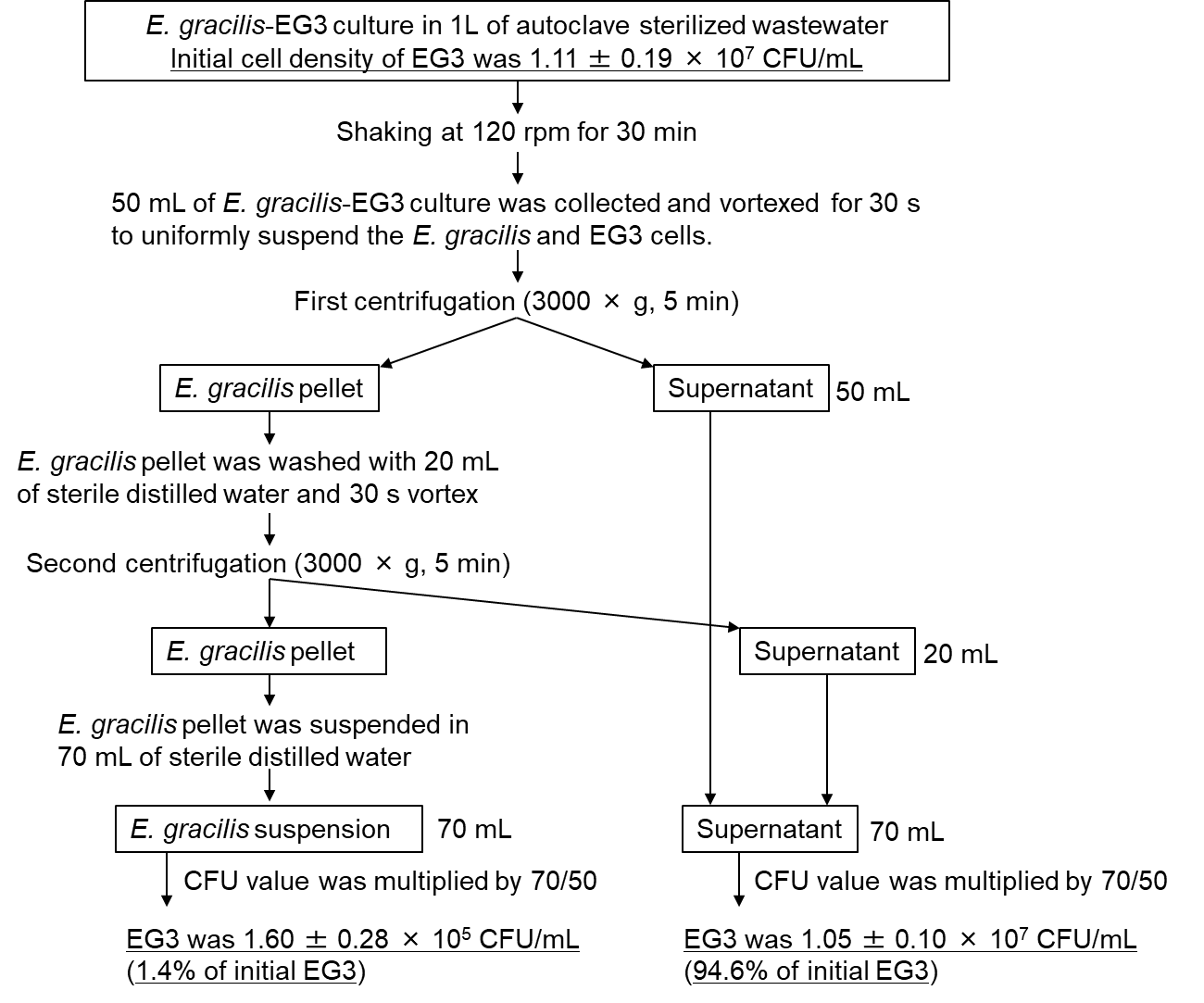


Fig. S1. Summary of *E. gracilis* collection method and fate of EG3 cells during this method.
