# Supplementary material for: Enhanced production of biomass and lipids by Euglena gracilis via co-culturing with a microalga growth-promoting bacterium, Emticicia sp. EG3
Source: Biotechnol Biofuels. 2019 Oct 31;12:205. doi: 10.1186/s13068-019-1544-2 (PMC6822413; doi:10.1186/s13068-019-1544-2)
Supplement: Supplementary file 2 — Additional file 2: Figure S2. Effects of isolated strains EG3, EG5, EG6, EG8, EG9, and EG10 on E. gracilis growth in autoclave heat-sterilized wastewater effluent. [file 13068_2019_1544_MOESM2_ESM.docx]

Additional file 2


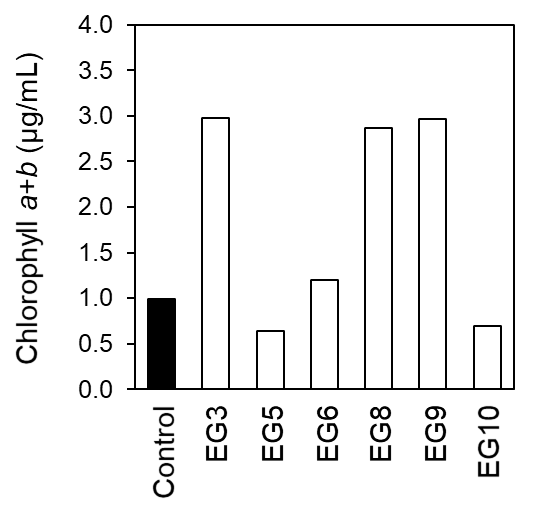


**Fig. S2.** Effects of isolated strains EG3, EG5, EG6, EG8, EG9, and EG10 on *E. gracilis* growth in autoclave heat-sterilized wastewater effluent. Chlorophyll *a*+*b* content of *E. gracilis* culture with and without (control) each strain in autoclaved effluent after 5 days.
